# Determinants of Vitamin D deficiency in asymptomatic healthy young medical students

**DOI:** 10.12669/pjms.345.15668

**Published:** 2018

**Authors:** Shabnam Nadeem, Tazeen Fatima Munim, Hareem Fatima Hussain, Deebaj Fatima Hussain

**Affiliations:** 1 Dr. Shabnam Nadeem, Assistant Professor, Gynae Unit-I, Karachi Medical & Dental College, Abbasi Saheed Hospital, Karachi, Pakistan; 2 Prof. Dr. Tazeen Fatima Munim, Head of the Department of Obstetrics/Gynaecology, Karachi Medical & Dental College, Abbasi Saheed Hospital, Karachi, Pakistan; 3 Dr. Hareem Fatima Hussain, House Surgeon, Dow University of Health Sciences (DUHS), Civil Hospital, Karachi, Pakistan; 4 Dr. Deebaj Fatima Hussain, House Surgeon, Dow University of Health Sciences (DUHS), Civil Hospital, Karachi, Pakistan

**Keywords:** Associated Risk Factors, Vitamin D, Young Healthy Medical Students

## Abstract

**Objective:**

The study aimed to determine vitamin D status and frequency of its determinants related to diet, clothing, lifestyle and exposure to sunlight among young healthy medical students living in Karachi, Pakistan.

**Methods:**

This cross sectional study included responses gathered on questionnaire from medical students of Karachi Medical &Dental College from 4^th^ of August 2017 till 30^th^ April 2018. All the participants were healthy young adult’s year of age who gave written informed consent to participate in the study. Questions regarding demographics, sun exposure, diet, clothing, living patterns and any symptoms like pain or body aches were recorded. Serum 25 (OH) vitamin D3, calcium and phosphorus levels were measured through laboratory examination. For serum 25OHD, the cutoff values ≤ 20ng /ml, ≥ 21-29ng /ml, and ≥ 30ng/ml were defined as deficiency, insufficiency, and sufficiency respectively.

**Results:**

Total number of medical students enrolled in the study was 221.Among total participants 191 (86.43%) were females and 30 (13.57%) were males. Mean ± SD age was almost similar (23.00 ± 2.56 vs. 23.03 ± 2.05). Majority of the females 115 (60.2%) had BMI within normal range, and were predominantly single 164 (85.9%). Vitamin D deficiency was found in 197 (89.14%), insufficiency in 16 (7.24%), and only 8 (3.62%) had sufficient levels. Determinant factors reported by deficient group (n=197); fabric color (dark mix) 87.3%, fabric material (synthetic + mix) 48.7%, full length of sleeves by 45.7%, exposure to sun only on hands and face during outdoors was reported by 64.5%, milk up to 250 cc was consumed by 77.2%, one egg per day in diet was taken by 56.9% and intake of cod liver oil was less common in only 27.4%.

**Conclusion:**

Deficiency of vitamin D is common among healthy young adults particularly females which emphasize need to add vitamin D supplements in their routine diet.

## INTRODUCTION

Vitamin D affects almost every organ system in the body. On exposure of skin to sun light rays, most of the vitamin D is synthesized naturally. Oily fishes contain small quantities of naturally occurring vitaminD.^1^ Vitamin D works as a hormone in the blood. It maintains the calcium and phosphate haemostasis which in turns promotes the healthy growth and remodeling of the bones. Vitamin D also has other effects, including cell growth, neuromuscular, immune modulations and improves tissue inflammation and tumor suppression.^2^ Synthesis of vitamin D is affected by latitude, environmental pollution, protecting clothing, melanin pigmentation, sun light exposure, dietary habits and malabsorption.^3^ Decreased levels of vitamin D increases the production of parathyroid hormone which leads to bone resorption and bone loss resulting in increased level of phosphorous and alkaline phosphatase.^4^ Vitamin D deficiency leads to rickets in children and osteomalacia and increase tendency of fracture occurs in adults and increased risk of common cancers, autoimmune diseases, hypertension, coronary artery disease, and infectious disease and cognitive disorders of brain.^5^

Vitamin D deficiency or insufficiency is highly reported in healthy children and adolescents and the frequency of this deficiency or insufficiency is increasing every year.^6^ A study was conducted in eastern province of Saudi Arabia among young healthy adults reported high prevalence of vitamin D deficiency.^7^ Another study conducted in Isfahan city of Iran reported 70.4% of healthy adults are vitamin D deficient.^8^ However, data on vitamin D deficiency in healthy young adolescent in our local population is still scarce. One study conducted in female medical students of Karachi showed high prevalence of vitamin D defeciency.^9^

Majority of individuals with vitamin D deficiency/insufficiency remain asymptomatic and it becomes difficult to detect it clinically. Complaints like muscular weakness and fatigue could be the clinical features associated with vitamin D deficiency but they are usually ignored and neglected. There are many contributing factors of vitamin D deficiency/Insufficiency in these individuals. In our society young girls as they cross their early childhood usually start wearing clothes cover most parts of the body which limit their exposure to sun. Uses of sun screen during travelling and sun bloc during outdoor activities, and living in multistoried buildings with no direct exposure to sun light are other contributing factors. Likes and dislikes of food items also contributes in vitamin D deficiency. Majority of children and adolescents are not directed to have proper diet, and dairy products like milk, yogurt, and eggs are lacking in their diet which makes them vitamin D deficient.

The paucity of information and health related issues associated with vitamin D deficiency has highlighted the need to investigate the vitamin D level in healthy young adults. The objective of study was to determine the levels of vitamin D and its association with possible determinant factors (demographic, lifestyle, dietary) among these subjects.

## METHODS

This was a descriptive, cross-sectional study, conducted at Gynae Unit I Department of Abbasi Shaheed Hospital from 4^th^ of August 2017 till 30^th^ April 2018. Ethical approval was obtained from Ethical and Scientific Review Committee of KM&DC. The medical students were recruited from Karachi Medical & Dental College, between 19-25 years of age by a non-probability convenient sampling technique. Subjects who were pregnant, lactating, had diagnosed metabolic bone disease, or systemic illness or were taking drugs affecting bone physiology, were not included in the study.

The sample size of n=221 calculation was done using the WHO software. The target sample size was achieved with 95% confidence level and 5% margin of error. After taking written informed consent from participants, data was collected through a questionnaire, followed by clinical examination and laboratory results.

This questionnaire was designed specifically to provide information about participant’s demographic characters, dietary habits, associated diseases and life style profile. The demographic characters included participant’s age, marital status, gender, profession and monthly income. Life style profile included clothing patterns, color and type of fabric, area of skin to sun exposure and its duration, use of sun bloc, mode of travelling, area of residence and its direction.

Dietary habits were assessed by daily intake of milk, egg with yolk, consumption of beverages, use of fish and cod liver oil. Clinical symptoms related to myalgia, fatigue, muscles cramps were included on questionnaire. The clinical examination included height, recorded in centimeters without shoes and weight recorded in kg without jackets, coats and abayas.

The laboratory investigations were collected by a phlebotomist from the participants in fasting state. The serum 25 (OH) D levels were measured to know the vitamin D status. The criteria for interpretation of vitamin D values is mentioned below.^10^

## 25 (OH) D LEVELS

Deficiency ≤20ng/ml
Insufficiency 21-29ng/ml
Sufficient ≥30ng/ml

The serum levels of calcium and phosphate were measured to evaluate the severity of vitamin D deficiency. Data analyzed from questionnaire, clinical examination findings and the laboratory results. Continuous variables were presented as mean ± standard deviations and median whereas categorical variables were presented in frequencies and percentages. The independent t test was used for normally distributed lab parameters (calcium & phosphate levels) and Mann Whitney U test used for non-normal distributed data of vitamin d levels. P-value <0.05 was considered statistically significant.

## RESULTS

Total number of participants was 221, of which 191 (86.43%) were females and 30 (13.57%) were males. Mean ± SD age of female and male medical students was similar (23.00 ± 2.56 vs. 23.03 ± 2.05). Majority of the respondents, 164 (85.9%) female and 29 (96.7%) male were single.

Vitamin D deficient group comprises of significant number of participants 197 (89.14%), insufficiency was reported in 16 (7.24%), and only 8 (3.62%) had sufficient levels. Mean (9.6 ± 8.7) vitamin D levels were reported in total number of participants. However, calcium (9.2 ± 0.89; n=185) and phosphate (4.15 ± 1.1; n=147) levels were within normal range for majority of the students (Table-I).

**Table-I:** Gender wise laboratory assessment of study participants.

Characteristics	Females (n=191)	Males (n=30)	P-value

	Mean ± SD (Median)		
Vitamin D Levels (ng/ml)	9.14 ± 8.89 (6.4)	12.80 ± 6.26 (12)	0.031*
Calcium Levels	9.15 ± 0.88 (9.1)	9.44 ± 0.88 (9.3)	0.096^∞^
Phosphate Levels	4.15 ± 1.15 (4)	4.13 ± 1.06 (4.1)	0.918^∞^

* Mann –Whitney U-Test,

∞ Independence sample t-test

Information on lifestyle factors showed that 87.3% of deficient students wear dark and mix color cloths, 48.7% wear synthetic and mix fabric and 45.7% of students wear full length sleeves. Exposure to sun of more than 30 minutes duration was more prevalent among 178 (90.4%) students, sun block was used by 68 (34.5%) students and students who had only face and hands exposed to sun were 127 (64.5%) which contributed more towards vitamin D deficiency. Majority of the participants were living in apartments 121 (61.4%) and 161 (81.7%) had west open houses and apartments (Table-II).

**Table-II:** Lifestyle and Dietary Factors among Vitamin D Deficient group (n=197)

Variable		N (%)
Fabric	***Color***	
	Dark + mix	172 (87.3)
	Light	25 (12.7)
	***Cloth***	
	Lawn	57 (28.9)
	Cotton	44 (22.3)
	Synthetic + mix	96 (48.7)
	***Length of Sleeves***	
	Quarter	14 (7.1)
	Half	14 (7.1)
	3/4	79 (40.1)
	Full	90 (45.7)
Exposure To Sun	***Duration***	
	<30mins	19 (9.6)
	>30mins	178 (90.4)
	***Use of Sun Block***	
	No	129 (65.5)
	Yes	68 (34.5)
	***Area of Skin Exposed***	
	Whole body covered	10 (5.1)
	Face exposure	11 (5.6)
	Face hand exposure	127 (64.5)
	Face hands & forearm exposure	49 (24.9)
Residence	***Type***	
	House	121 (61.4)
	Apartment	76 (38.6)
	***Covered square feet***	
	80-200 Yards	102 (51.8)
	201-400 Yards	63 (32)
	401-600 Yards	29 (14.7)
	601-1000 Yards	3 (1.5)
	***Direction***	
	East	36 (18.3)
	West	161 (81.7)
Diet	***Consumption of Beverages***	
	Tea	100 (50.8)
	Coffee	5 (2.5)
	Cold Drink	12 (6.1)
	Mix	64 (32.5)
	Nil	16 (8.1)
	***Intake of Milk/day***	
	Up to 250cc	152 (77.2)
	500 cc	18 (9.1)
	750 cc	2 (1)
	Nil	25 (12.7)
	***Consumption of Egg/day***	
	Once	112 (56.9)
	Twice	4 (2)
	None	81 (41.1)
	***Intake of cod liver oil***	
	No	143 (72.6)
	Yes	54 (27.4)

Factors related to diet included intake of milk, egg and cod liver oil as food sources for vitamin D. Consumption of milk per day of up to 250 cc was reported by 152 (77.2%), one egg per day in diet was taken by 112 (56.9%), and intake of cod liver oil was less common in only 54 (27.4%) (Table-II). Total 113 (59.40%) of medical students had BMI within normal range (18.5-25 kg/m^2^), 21 (10.70) of medical students were obese and 34 (17.30% of medical students) were overweight.

The symptom of body ache was reported by 59 students (29.9%), GERD by 22 students (11.2%) and allergy by 37 students (18.8%) in the vitamin D deficient group.

## DISCUSSION

This study was focused on an affluent and healthy population having no financial barriers to take healthy diet containing vitamin D but their vitamin D levels was found to be low. Nearly ninety percent of the subjects were vitamin D deficient. These findings were consistent with local studies by Shireen M et al. and Fatima et al. reported ninety-nine percent and ninety-five percent of subjects were vitamin D deficient.^4^,^9^ Our findings are also supported by many studies from South East Asia and Middle East on vitamin D levels in recent years.^8^,^11^

The majority of participants with decrease levels of vitamin D were female and unmarried. This is alarming as these are potential child bearing women. Heckmatt JZ et al. has shown that low levels of vitamin D in pregnancy lead to poor pregnancy outcome (neonatal hypocalcaemia, rickets and enamel hypoplasia of primary dentition in infants.^12^

It is interesting that seventy-seven percent of vitamin D deficient subjects took a glass of milk and fifty-seven percent ate one egg per day indicating that other possible factors may be involved. A very interesting observation was that majority of vitamin D deficient subjects took sugar sweetened beverages like tea, soft drink and juices. Limited studies have been done to evaluate the association between intake of sugar sweetened beverages and of vitamin D status. A recent study was conducted by Duchaine CS et al.^13^ who reported strong association of deficiency of vitamin D with high intake of cold drink in pre-menopausal women.^14^ Another study was conducted by Olson and colleagues who reported that high intake of soda and juices were associated with vitamin D deficiency.^14^ Our observations are consistent with these studies.

Our study shows conflicting results about the effect of exposure to sun on vitamin D deficiency. Bonilla C et al. has shown an association of color of clothing with vitamin D levels.^15^ Our study is consistent with this finding, most of vitamin D deficient subjects liked to wear dark color cloths. It is very surprising that ninety percent of the vitamin D deficient subjects had>30 minutes of sun exposure. This is in contrast with the study conducted in Denmark by Hensen L et al. who reported low level of vitamin associated with decrease sun exposure by using shades and wearing protective clothes during outdoor activities.^16^ According to our study results, eighty percent of vitamin D deficient subjects had no known allergies. This is in contrast with the study conducted in Korea by Jung JW and colleagues who reported a correlation between vitamin D and allergic diseases.^17^

Another interesting finding in our study was that 59% of vitamin D deficient subjects had normal BMI. This is in contrast with the study was conducted by Zhang HQ et al on Chinese school children who reported decrease levels of vitamin D with high BMI.^18^

### Limitations of the study

This study cannot be generalized to the entire population of youths. It includes small sample size due to financial burden of funding. It would be more beneficial to measure BMD in the subjects to see the effects of bone mineralization. It would also be necessary to check parathyroid hormone levels in vitamin D deficient subjects to rule out secondary hyperparathyroidism because serum calcium, phosphorous and alkaline phosphates were normal levels among vitamin D deficient subjects.

## CONCLUSION

Our study concludes that vitamin D deficiency/insufficiency is common in asymptomatic, educated healthy young medical students of Karachi. Despite the fact they have good sun exposure and took calcium containing diet.

### Recommendations

Vitamin D supplements should be recommended in all asymptomatic healthy young adults in their routine diet.

### Importance of study

To diagnose and treatment of vitamin D deficiency/Insufficiency in healthy young adults especially females could reduce vitamin D deficiency in newborn babies and osteomalacia in children and ultimately would reduce the risk of cardiovascular diseases, diabetes, cancers and in this way we could reduce the socioeconomic burden of society.

### Author`s Contribution

**SN:** Conception and Design.

**HF and DF:** Interpretation of data.

**SN:** Data analysis.

**TFM:** Final approval of the version to be published.
